# Associations of participation in organized sports and physical activity in preschool children: a cross-sectional study

**DOI:** 10.1186/s12887-020-02222-6

**Published:** 2020-07-02

**Authors:** Chu Chen, Fanny Sellberg, Viktor H. Ahlqvist, Martin Neovius, Filip Christiansen, Daniel Berglind

**Affiliations:** 1 Center for Epidemiology and Community Medicine, Region Stockholm, Solna vägen 1E, Stockholm, Sweden; 2 Department of Global Public Health, Karolinska Institutet, Solna, Sweden; 3 Clinical Epidemiology Division, Department of Medicine (Solna), Karolinska Institutet, Solna, Sweden

**Keywords:** Exercise, Sedentary, Sports, Preschool, Accelerometer

## Abstract

**Background:**

Participation in organized sports is associated with higher physical activity (PA) levels in school-aged-children. Yet, little is known about PA determinants in preschool-aged-children. We examined associations between organized sports participation and preschoolers’ daily PA.

**Methods:**

The study comprised 290 3–5 years old children and PA was measured for 1 week via accelerometers. Organized sports participation was parent-reported and preschool arrival and departure time was teacher-recorded. The preschool duration reported by teachers was matched with time-stamped accelerometer data to distinguish PA during preschool time and PA outside preschool time. Linear mixed models, nested on preschool level, were used to examine associations between organized sports participation and children’s PA outside preschool time, during preschool time and throughout the day.

**Results:**

In total, 146 children (50.3%) participated in organized sports at least 1 h/week. Participation in organized sports was associated with 6.0 more minutes of moderate-to-vigorous PA (MVPA) (95% CI: 0.6, 11.3) throughout the day and 5.7 more minutes of MVPA (95% CI: 1.6, 9.7) outside preschool time after adjustment. There was no association between organized sports participation and PA during preschool time.

**Conclusions:**

This is the first study to show positive associations between organized sports participation and preschoolers’ PA levels outside preschool time and throughout the day. In addition, findings from this study do not support PA compensation. Therefore, targeting organized sports may be successful in improving PA, even among preschoolers.

## Introduction

There are well-established associations between total physical activity (PA), moderate to vigorous PA (MVPA) [1], steps [2] and both short- and long-term health benefits in children, while more conflicting findings have been reported for objectively measured sedentary time (ST) [3]. Beyond the immediate early life (0–4 years) health benefits associated with regular PA, physically active children also have a tendency to continue being physically active across their lifespan [4]. Therefore, a major concern is the low levels of PA among young children [5]. According to a review on preschoolers’ objectively measured physical activity level, preschoolers spend 2–41% of their day in MVPA, 4–33% in light PA (LPA), and 34–94% sedentary [6]. Moreover, Swedish data with objectively measured PA, show that preschool aged children’s PA levels are low, especially outside preschool hours and on weekend days [7].

Organized sports, which is defined as *“PA that is directed by adult or youth leaders and involves rules and formal practice and competition”* [8], have been associated with reduced risk of morbidity. In adolescents, participation in organized sports is associated with higher levels of PA [9], decreased risk for cardiovascular disease [10], improved mental health and social adjustment [11]. Thus, participation in organized sports is a potential strategy to improve adolescents’ physical and mental health by increasing their PA [9–11]. Moreover, childhood exposure to organized sports may be influential on participation in later years [12]. However, little is known about the association between organized sports participation and PA levels in preschool aged children [13]. Specifically, it has been argued that organized sports has preliminary health benefits for preschool children, but no previous study have addressed PA level as an outcome and objective measure of outcomes in this young age group is lacking [14]. Further, organized sports participation have been described as a behavioral determinant of PA, despite that definite conclusions have been hampered by the limited number of studies [15]. For example, a previous review identified only two studies on this topic but neither study applied standardized objective measures of PA [15]. In addition, while MVPA has established positive associations with numerous health outcomes particularly for preschool children, LPA is not as beneficial [1]. Previous studies without the employment of accelerometry to classify PA intensity levels fail to identify the PA pattern which may be associated with participation in organized sports. Further studies that distinguish the PA patterns are imperative to understand the possible benefits of organized sports participation. Despite this, early life entry into organized sports is increasingly being recommended due to the inactivity level of young children and the high dropout rate in organized sports in older children [16].

There are studies showing a PA synergy, where participation in exercise/PA increases PA at other times of the day [17, 18]. However, there is a conflicting “activity-stat” hypothesis, which proposes that children compensate for increased PA in during part of the day (e.g. during organized sports participation) by decreasing their PA at another time (e.g. during preschool time); thus, maintaining a fixed level of total PA [19]. This hypothesis is supported by review data indicating that approximately 63% of exercise/PA intervention studies, that specifically examined this hypothesis in children, have reported PA compensation [20]. If preschool children maintain a fixed level of total PA despite engaging in organized sports, any interventions targeting organized sports may be ineffective in achieving health benefits associated with PA. The aforementioned review study also calls for more evidence to examine the validity of the hypothesis [20]. In preschool children, the compensatory behavior was not observed in studies comparing days with and without teacher-led structured physical activity [21], comparing between locations (childcare center and home) [22], and when increasing outdoor time [23]. Yet, to the best of our knowledge, no previous study has investigated the association between participation in organized sports and preschool children’s activity levels in different segments of the day.

Based on these knowledge gaps, the aims of the current study were to examine associations between participation in organized sports and objectively measured PA and ST: (i) outside preschool time, (ii) during preschool time and (iii) throughout the day in a sample of Swedish 3–5 years old children.

## Methods

### Study setting, design, and population

Within the Södermalm district of Stockholm Sweden, a convenience sample of 30 out of the total 51 municipal preschools were invited to participate in the current cross-sectional observational study. Out of 30 preschools invited, 27 preschools chose to participate, including a total of 1178 children. At the participating preschools, all children between 3 and 5 years of age were invited to participate. Out of the 1178 total children, 405 (23%) children and their parents consented to participation in the study. In Sweden, children aged below 6 attend preschools every weekday (i.e. 5/7 in a week). The study was approved by the Stockholm Ethical Review Board (Dnr: 2018/890–31/2), and informed consent was obtained from participating children’s parents and preschool teachers. The fieldwork measurements, including body measures of children, 7 days of accelerometer measures of PA in children and parental questionnaires, were carried out during September to November 2018.

### Participation in organized sports (exposure)

Parents to participating children filled in a questionnaire with the question “Does your child participate in any kind of organized sports?”. The answer options were (i) no participation, (ii) 1–2 h organized sports/week, (iii) 3 h organized sports/week or (iv) 4 or more hours organized sports/week. Organized sports are generally accepted as structured leisure time activities in non-profit organization [24]. Therefore, the participation in organized sports assessed here was explicitly about sports participation outside preschool hours. Due to the low participation in organized sports of more than 2 h/week (10%), we classified participation in organized sports into a dichotomous variable: (i) No organized sports and (ii) at least one-hour participation in organized sports/week.

### Physical activity and sedentary time outside preschool time, during preschool time and throughout the day (outcomes)

The outcome measures, PA and ST outside preschool time, during preschool time and throughout the day, were measured via the triaxial Actigraph GT3X+ accelerometer, which has been widely used to assess PA and ST in pediatric research [25]. We consulted best practices for wear protocol and analysis [25] and as such decided our procedure to be the following: All children were instructed to wear accelerometer, at right hip for 7 consecutive days, during all waking hours and children that had worn the accelerometer for at least 3 days with 10 or more wear-time hours per day were included in the analytical dataset [25]. Non-wear time was defined as 60 or more consecutive minutes with zero counts, allowing up to 2 min of interruptions with non-zero counts [26] after adaptation for a potentially less compliant sample (preschool children) [27]. We analyzed vector magnitude (V_m_) activity counts (V_m_ = √ (X^2^ + Y^2^ + Z^2^)) in 60s epochs following the calibration study by Butte et al. that developed MVPA, LPA and ST cut-offs specifically for V_m_ activity counts in 4-year old children [28]: ST was calculated as any minute of less than 820 counts per minute (cpm), LPA as 820–3907 cpm and MVPA as ≥3908 cpm. Steps were determined using the manufacturer’s step algorithm, using the normal filter setting.

During the 7 days of accelerometer measures in children, preschool staff recorded time arrival to and leaving the preschool on daily basis for each participating child. This data was thereafter matched, on daily level, to time-stamped accelerometer data which enabled us to calculate PA and ST before, after and during preschool. PA and ST before and after preschool time were then combined with PA and ST during the weekend to calculate PA and ST outside preschool time. PA and ST throughout the day was considered as all wear-time hours during the day. The mean daily PA and ST outside preschool time, during preschool time and throughout the day were calculated on the individual level and then matched with data on organized sports participation.

### Covariates

Anthropometry and family characteristics were also documented. Weight and height of participating children were measured using validated scales and stadiometers, respectively (scale: VB2–200-EC, Vetek AB, Väddö, Sweden, stadiometer: Seca 213, Seca, Chino, CA, USA). We used an age and sex specific international body mass index (BMI) classification by Cole et al. [29] to classify children as normal weight, overweight or obese. Parents filled out a questionnaire on demographical variables such as number of siblings within the family and highest parental education level, categorized into elementary school, upper secondary school and university education.

### Statistical analysis

Appropriate measures of variability and central tendency, mean and standard deviation (SD) for normally distributed variables or median and interquartile range (IQR) for variables with skewed distribution, are presented for various background characteristics and PA outcomes of the total study population and stratified by participation in organized sports.

We used linear mixed models, nested on preschool level, to examine associations between participation in organized sports with children’s daily levels of MVPA, LPA, steps and ST outside preschool time, during preschool time and throughout the day. Separate models were fitted for each activity intensity. In addition, we performed robust multilevel mixed-effects Poisson regression, nested on preschool level, to examine the likelihood of meeting the current World Health Organization (WHO) PA guidelines for children under five [30], of at least 60 min of daily MVPA, in organized sports participation with no participation in organized sports as reference level. All models were adjusted for confounders, factors that potentially influences both exposure (participation in organized sports) and outcomes, selected based on causal reasoning [31]. The selected confounders were age of the children, sex, overweight/obesity status [32], accelerometer wear-time [33], number of siblings [34] and parental education [35]. We calculated all *p*-values for both Poisson and linear mixed models using Wald tests, testing the coefficient of interest being equal to zero. To test for sex-specific associations, we performed a Wald test of the coefficient of a product interaction between sex and organized sports participation and stratified our main analysis.

In sensitivity analyses, we compared the descriptive characteristics between the analytical dataset (*n* = 290) and excluded observations (*n* = 104).

Raw accelerometer data was processed in Actilife version 6.13.3 and all statistical analysis were conducted in Stata version 14.2.

## Results

**Fig. 1:**
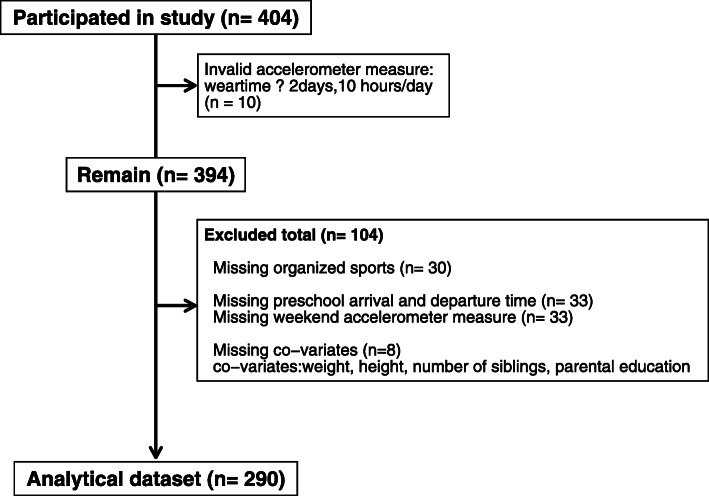
Flow chart of participants. Description of participants included in the analytical data set (*n* = 290) and excluded participants (*n* = 114)

In total, 404 children aged 3–5 years participated (Fig. 1). Among participants, 114 children were excluded with 10 children disqualified due to invalid accelerometer data and 104 children excluded due to missing value. Of the children with missing value, we first excluded 30 children who had missing data on organized sports participation, secondly the 33 children who had missing data on preschool arrival and departure time, thirdly the 33 children who did not have weekend accelerometer data to determine PA outside preschool time, and finally 8 children who had missing co-variate data (weight, height, number of siblings and parental education). Consequently, the final analytical sample comprised 290 children with valid accelerometer data, data on organized sports participation, time outside/at preschool and all co-variate variables.

Table 1 displays an overview of descriptive characteristics and children’s levels of PA (outcomes) by participation in organized sports (exposure) in categories of no participation in organized sports versus participation in at least 1 h of organized sports/week. In total, 146 children (50.3%) participated in organized sports at least 1 h/week. Of those children who participated in organized sports, 131 (90%) participated 1–2 h/week, 12 (8%) 3 h/week and 3 (2%) 4 or more hours/week.

**Table 1:** Descriptive characteristics in relation to participation in organized sports

	**Participation in organized sports**		
	**No (*n*** **= 144)**	**Yes (*n*** **= 146)** **At least 1 h per week**	**Total (*n*** **= 290)**
**Descriptive characteristics**			
Girls, n (%)	67 (46.5%)	64 (43.8%)	131 (45.2%)
Age, mean (SD)	4.4 (0.8)	5.0 (0.6)	4.7 (0.8)
Overweight/obese, n (%)	11 (7.6%)	16 (11.0%)	27 (9.3%)
Number of siblings,	1.0 (0.8)	1.1 (0.7)	1.1 (0.8)
0, n (%)	33 (22.9%)	22 (15.7%)	55 (19.0%)
1, n (%)	91 (63.2%)	88 (60.3%)	179 (61.7%)
2, n (%)	16 (11.1%)	33 (22.7%)	49 (16.9%)
≥ 3, n (%)	4 (2.8%)	3 (2.3%)	7 (2.4%)
Parental education,			
Elementary school, n (%)	2 (1.4%)	3 (2.0%)	5 (1.7)
Secondary school, n (%)	29 (20.1%)	21 (14.4%)	50 (17.3%)
University, n (%)	113 (78.5%)	122 (83.6%)	235 (81.0%)
**Physical activity outside preschool time, mean (SD)**			
Moderate-to-vigorous physical activity (min)	31.3 (14.6)	40.8 (18.8)	36.1 (17.5)
Light physical activity (min)	220.5 (37.2)	225.5 (41.5)	223.1 (39.5)
Steps (counts)	5510 (1427)	6627 (1663)	6072 (1646)
Sedentary time (min)	270.3 (61.1)	266.0 (72.3)	268.2 (66.9)
Wear-time (min)	522.2 (68.2)	532.4 (71.1)	527.3 (69.7)
**Physical activity during preschool time, mean (SD)**			
Moderate-to-vigorous physical activity (min)	32.3 (20.1)	40.6 (20.5)	36.5 (20.7)
Light physical activity (min)	247.0 (49.5)	240.9 (40.1)	243.9 (45.1)
Steps (counts)	6684 (2185)	7161 (2108)	6924 (2156)
Sedentary time (min)	172.1 (43.0)	161.5 (40.2)	166.7 (41.9)
Wear-time (min)	451.5 (66.2)	443.1 (50.7)	447.3 (59.0)
**Physical activity whole day, mean (SD)**			
Moderate-to-vigorous physical activity (min)	47.1 (21.3)	60.6 (25.3)	53.9 (24.3)
≥ 60 min/day, n (%)	38 (26.4%)	64 (43.8%)	102 (35.2%)
Light physical activity (min)	350.9 (43.4)	355.3 (42.1)	353.1 (42.7)
Steps (counts)	9053 (2061)	10,397 (2134)	9730 (2200)
Sedentary time (min)	355.2 (64.7)	342.6 (66.3)	348.8 (65.7)
Wear-time (min)	753.2 (55.5)	758.5 (56.8)	755.9 (56.1)

*Abbreviation*: *SD* standard deviation

**Fig. 2:**
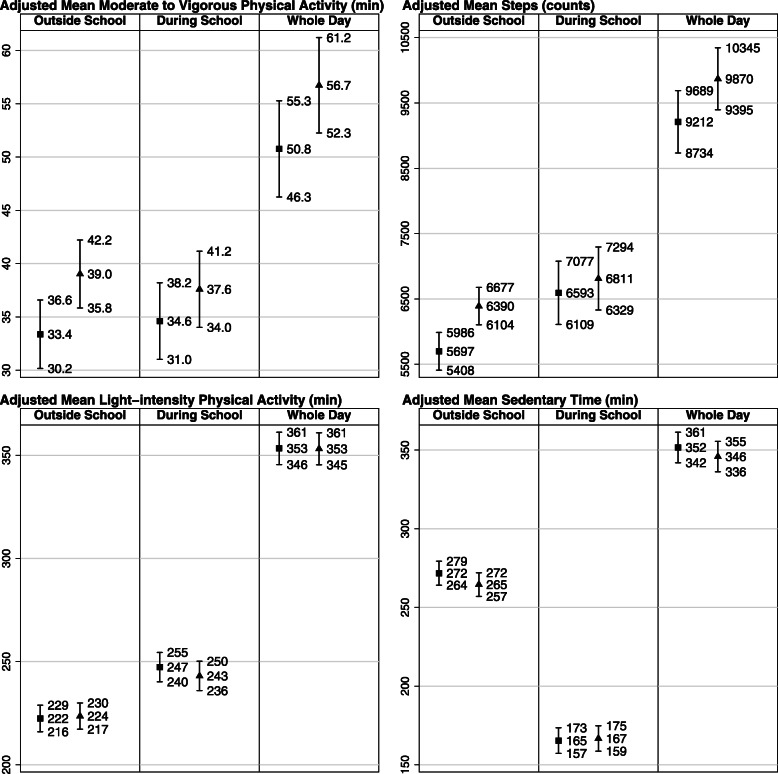
Associations between participation in organized sports and moderate-to-vigorous physical activity, steps, light physical activity and sedentary time outside preschool time, during preschool time and throughout the whole day. Adjusted for sex, age, overweight/obesity status, accelerometer wear-time, parental education and number of siblings. Square: not participating in organized sports. Triangle: participating in organized sports

Figure 2 illustrates associations between participation in organized sports and MVPA, LPA, steps and ST outside preschool time, during preschool time throughout the whole day in adjusted analyses. Children who participated in organized sports took 693 more steps (95% CI: 331, 1056; *p* < 0.001) outside preschool time and 658 more steps (95% CI: 237, 1079; p < 0.001) throughout the whole day compared with children who did not participate in organized sports (Additional file 1, Additional Table 1). Similarly, children participating in organized sports spent 5.7 more minutes in MVPA (95% CI: 1.6, 9.7; *p* = 0.03) outside preschool time and 6.0 more minutes in MVPA (95% CI: 0.6, 11.3; *p* = 0.01) throughout the whole day. There were no associations between participation in organized sports and steps (*p* = 0.20) or MVPA (*p* = 0.18) during preschool time. Although the interaction between sex and organized sports participation was not statistically significant (*P* > 0.05), associations between participation in organized sports and all PA indicators outside preschool time, during preschool time and throughout the whole day were stronger in boys compared to girls (Additional file 1, additional Table 1).

Additional Table 2 in Additional file 1 illustrates associations between participation in organized sports and meeting the PA recommendation, of 60 or more minutes of daily MVPA, in crude and adjusted analyses. In adjusted analysis, participation in organized sports was associated with a 20% higher likelihood of meeting the daily PA recommendation (RR 1.2, 95% CI: 0.8, 1.9). However, this association was not statistically significant (*p* > 0.05).

Additional Table 3 in Additional file 1 provides an overview of descriptive characteristics and child PA indicators throughout the whole day between the analytical dataset (*n* = 290) and excluded observations (*n* = 104). On average, participants in the analytical dataset and those excluded from analyses were similar with regards to descriptive characteristics and PA throughout the day.

## Discussion

The current study was the first to examine associations between participation in organized sports and objectively measured PA and ST outside preschool time, during preschool time and throughout the day in preschool-aged children. Our findings showed that approximately 50% of children participated in organized sports for 1 or more hours/week. Participation in organized sports was positively associated with children’s MVPA and steps outside preschool time and throughout the whole day. There was no association between participation in organized sports and PA during preschool time. Thus, findings from the current study do not support a PA compensation in preschool children participating in organized sports.

Studies in school aged children and adolescents have shown a positive association between participation in organized sports and children’s accelerometer measured PA throughout the day [36–38]. Previous studies have also shown a significant interaction between organized sports participation and sex on children’s PA [37–39]. Results from the current study showing that organized sports participation was associated with 6.0 min (10%) more MVPA throughout the day, and potentially a greater association in boys compared with girls, are in line with those presented in above-mentioned studies. The activity-stat hypothesis is supported by several exercise intervention studies in older children (school aged), showing a compensation in PA [20]. In contrast, results from the current study show that preschoolers who participate in organized sports are equally active as those who do not participate in organized sports during preschool time and more active both outside preschool time and throughout the whole day.

Given the low and decreasing levels of PA among young children [5, 40], it is important to find effective strategies to increase young children’s PA. Meta-analyses data of trials examining the effectiveness of interventions to increase PA in children show minor treatment effects; adding up to approximately 4 min of additional walking or running per day [41]. Although the results from this cross-sectional study cannot be compared to results from that of interventions, the 6.0 min (10%) more MVPA associated with participation in organized sports is a practically significant difference which may warrant further intervention studies of organized sports in preschool children. Although intensity of PA is of great importance, benefits of participation of organized sports for preschool children during this particular fast developing and maturation stage may be considered in a broader spectrum [42]. Organized sports programs in preschool age are usually tailored for their development stage and aims not only to increase PA level, but also to strive for various beneficial developmental outcomes such as social skills and self-regulation or simply emphasize the “fun in activity” for children [43]. Especially young children would spend longer time to understand the rules, learn to play with other children and acquire skills to be able to handle more complex physical movement. These complex and interacting benefits associated with participation in organized sport in this age group may, beyond the direct effects of sports participation, explain the increased MVPA associated with organized sports in preschoolers. This finding, in combination with the positive effect participation in organized sports may have on the tracking of PA from childhood to adulthood [4], supports early life participation in organized sports as a strategy to achieve higher levels of PA throughout childhood, adolescence and adulthood. The higher PA level, potentially resulted from participation in organized sports, can further benefit a wide range of health indicator in preschool children comprising bone strength, motor development, fitness and psychosocial health [1]. However, the observational and cross-sectional nature of the current study can only provide evidence of a positive association between organized sports participation and PA throughout the day in preschoolers. A randomized controlled trial in preschool aged children is warranted to examine if the associations observed in the current study hold.

Apart from higher MVPA, children participated in organized sports had 658 more steps throughout the whole day. Step counts is a valid way to record PA in the preschool population [44], but it is not generally discussed in accelerometry-based study because there is no standardized classification of PA levels based on steps [45, 46]. However, accelerometry is relatively expensive and requires specialized software for initialization of accelerometers and data analysis which often is not user friendly to the general public. In contrast, steps information can be easily measured via pedometers or mobile digital devices (e.g. smartphones). In addition, an increase of 658 steps/day associated with organized sports participation is possibly easier to understand than 6 more minutes MVPA/day to parents, teachers and coaches who may influence preschool children’s participation in organized sports. Therefore, steps data offers unique opportunity to understand, monitor preschool children’s PA and disseminate research findings. Study results in terms of steps should not be neglected and further PA research to standardize the handling of steps data is warranted.

A strength of this study was the accelerometer measured PA which limit several biases associated with self-reported measures, e.g. social desirability and recall difficulties. Second, the time-stamped accelerometer PA data and detailed daily data on time at and outside preschool enabled us to assess the association between organized sport participation and PA levels during preschool time, outside preschool time and throughout the day. Third, in the aspect of obtaining organized sports participation information and objectively measured PA data, the current study included a fairly large number of children in a previous unstudied age group.

Limitations of our study include that information on organized sports participation relied on parental self-reporting, which has several inherent biases and we did not take type of sport performed into account. Second, the observational and cross-sectional nature of the study preclude any inference about causality, and we cannot rule out that our results may be explained by unmeasured confounders or reverse causality, i.e. children who are more active chose to participate in organized sports and not vice versa. Third, although accelerometry is considered as a desirable measurement of PA among preschool aged children in free-living conditions, hip-worn accelerometers are unable to detect some types of PA, e.g. cycling, swimming or PA involving upper body movements [47]. Further, the inability to capture PA performed during certain activities (e.g. swimming) would lead to an underestimation of PA. As such activities may be more common in children participating in organized sport, it could be argued that the association between organized sport and PA shown in this study may be a conservative estimate. In addition, it is possible that daytime napping may influence the estimation of ST and could have contributed to excessive elimination of participants due to insufficient wear time if napping time was classified as non-wear time. However, napping is usually not a scheduled activity in Swedish preschool [48] and all children with invalid accelerometer data was eliminated due to less than 3 days of accelerometer data rather less than 10 h/day wear time. Therefore, unrecorded napping time during the day may have limited influence in this study. Forth, by using the normal filter to process accelerometer data we may have underestimated children’s number of steps taken per day. In addition, accelerometer data were analyzed in 60s epoch while a shorter epoch-length of analysis have been suggested to suit the children’s sporadic moving nature [25]. However, tailoring the cut-offs to suit a different epoch may cause some problems [49]. The cut-offs of PA level classification may be most accurate when they are applied under the same condition as the calibration study that developed these cut-offs [49, 50]. Therefore, accelerometer data was analyzed in 60 s epoch strictly following the epoch setting in the calibration study of PA level cut-offs [28]. Fifth, the sample of preschool children was selected based on acceptance of the invitation from the invited children and parents which could potentially influence the generalizability of the study. Finally, the study population was homogenous in terms of socio-economic aspects and urbanity. This is of importance as participation in organized sports may be associated with household finances due to high costs for many of these activities. Consequently, our results have limited generalizability to rural and lower socioeconomic areas.

## Conclusions

To the best of our knowledge, the current study is the first to show, in preschool children, a positive association between participation in organized sports and objectively measured moderate-to-vigorous physical activity and steps outside preschool time and throughout the whole day. There was no association between participation in organized sports and physical activity during preschool time. Thus, results from the current study do not support PA compensation among preschool aged children. Targeting organized sports may be successful in improving PA, even among very young children (3–5 years old). This could contribute to forming interest and habit of sport participation from early age and throughout childhood [4] with a wide range of benefits such as improving fitness, motor development and psychosocial health [1].

## Supplementary information

**Additional file 1: Table S1.** Associations between participation in organized sports and physical activity indicators outside preschool time, during preschool time and throughout the whole day. **Table S2.** Associations between participation in organized sports and likelihood of meeting the physical activity recommendation. **Table S3.** Comparison of descriptive characteristics between analytical dataset and excluded observations.

## Data Availability

The datasets used and/or analysed during the current study are available from the corresponding author on reasonable request.

## Abbreviations

IQR: Interquartile range
LPA: Light physical activity
MVPA: Moderate to vigorous physical activity
PA: Physical activity
SD: Standard deviation
ST: Sedentary time
WHO: World health organization
