# Supplementary material for: Associations of participation in organized sports and physical activity in preschool children: a cross-sectional study
Source: BMC Pediatr. 2020 Jul 2;20:328. doi: 10.1186/s12887-020-02222-6 (PMC7331172; doi:10.1186/s12887-020-02222-6)
Supplement: Supplementary file 1 — Additional file 1: Table S1. Associations between participation in organized sports and physical activity indicators outside preschool time, during preschool time and throughout the whole day. Table S2. Associations between participation in organized sports and likelihood of meeting the physical activity recommendation. Table S3. Comparison of descriptive characteristics between analytical dataset and excluded observations. [file 12887_2020_2222_MOESM1_ESM.docx]

|  | **Outside preschool time** | | | | |  | **During preschool time** | | | | |  | **Throughout the whole day** | | | | |
| --- | --- | --- | --- | --- | --- | --- | --- | --- | --- | --- | --- | --- | --- | --- | --- | --- | --- |
|  | **Crude model** | |  | **Adjusted model** | |  | **Crude model** | |  | **Adjusted model** | |  | **Crude model** | |  | **Adjusted Model** | |
|  | Coef. | 95% CI |  | Coef. | 95% CI |  | Coef. | 95% CI |  | Coef. | 95% CI |  | Coef. | 95% CI |  | Coef. | 95% CI |
| **All (n=290)** |  |  |  |  |  |  |  |  |  |  |  |  |  |  |  |  |  |
| MVPA (min) | 8.5 | 4.6, 12.4 |  | 5.7 | 1.6, 9.7 |  | 6.9 | 2.3, 11.6 |  | 3.0 | -1.4, 7.4 |  | 11.9 | 6.5, 17.3 |  | 6.0 | 0.6, 11.3 |
| LPA (min) | 4.8 | -4.3, 14.0 |  | 1.2 | -8.0, 10.4 |  | -6.3 | -15.5, 2.8 |  | -4.3 | -11.2, 2.6 |  | 3.9 | -6.1, 13.8 |  | -0.2 | -10.4, 10.0 |
| Steps (counts) | 987 | 634, 134 |  | 693 | 331, 1056 |  | 401 | -1, 804 |  | 218.3 | -114, 551 |  | 1141 | 696, 1586 |  | 658 | 237, 1079 |
| ST (min) | -4.6 | -20.1, 11.0 |  | -7.2 | -17.5, 3.1 |  | -6.2 | -15.0, 2.7 |  | 1.4 | -7.0, 9.7 |  | -11.3 | -26.5, 4.0 |  | -5.8 | -17.8, 6.2 |
| **Boys (n=159**) |  |  |  |  |  |  |  |  |  |  |  |  |  |  |  |  |  |
| MVPA (min) | 8.8 | 3.5, 14.2 |  | 5.5 | -0.5, 11.5 |  | 10.4 | 3.7, 17.1 |  | 4.9 | -2.2, 12.0 |  | 14.5 | 6.7, 22.3 |  | 7.7 | -0.6, 16.1 |
| LPA (min) | 3.8 | -9.0, 16.7 |  | 2.7 | -11.0, 16.5 |  | -8.0 | -20.3, 4.3 |  | -7.4 | -17.1, 2.3 |  | 2.3 | -11.0, 15.7 |  | 3.0 | -11.3, 17.3 |
| Steps (counts) | 1087 | 616, 1557 |  | 693 | 192, 1194 |  | 753 | 235, 1272 |  | 516 | 42, 991 |  | 1439 | 859, 202 |  | 930 | 341, 152 |
| ST (min) | -2.2 | -22.7, 18.4 |  | -8.4 | -23.7, 6.8 |  | -4.9 | -16.7, 6.9 |  | 2.4 | -9.5, 14.2 |  | -9.9 | -29.4, 9.6 |  | -10.7 | -27.6, 6.3 |
| **Girls (n=131)** |  |  |  |  |  |  |  |  |  |  |  |  |  |  |  |  |  |
| MVPA (min) | 7.8 | 2.2, 13.3 |  | 5.3 | -0.2, 10.8 |  | 2.9 | -3.0, 8.8 |  | 0.8 | -4.4, 6.0 |  | 8.7 | 1.8, 15.6 |  | 4.4 | -2.2, 11.0 |
| LPA (min) | 6.3 | -6.6, 19.1 |  | 1.1 | -10.9, 13.0 |  | -2.7 | -16.7, 11.3 |  | 0.2 | -10.0, 10.4 |  | 5.6 | -9.1, 20.3 |  | -0.4 | -14.6, 13.8 |
| Steps (counts) | 890 | 351, 1429 |  | 649 | 117, 1182 |  | -42 | -686, 602 |  | -130 | -601, 341 |  | 761 | 71, 1451 |  | 330 | -285, 946 |
| ST (min) | -10.4 | -34.0, 13.1 |  | -7.6 | -21.2, 6.0 |  | -6.2 | -19.4, 6.9 |  | -0.6 | -12.9, 11.7 |  | -14.2 | -38.0, 9.6 |  | -4.5 | -21.3, 12.3 |
| **Additional Table 1 Associations between participation in organized sports and physical activity indicators outside preschool time, during preschool time and throughout the whole day**  Reference level: no participation in organized sports  Adjusted model: adjusted for sex, age, overweight/obesity status, accelerometer wear-time, parental education and number of siblings  Abbreviations: PA = physical activity, MVPA = moderate to vigorous physical activity, LPA = light physical activity, ST = sedentary time, CI = confidence interval | | | | | | | | | | | | | | | | | |

| **Participation in organized sport** | **Meet moderate to vigorous physical activity ≥ 60min/day, No. cases (%)** | **Risk ratio (95% CI)** | | **p-value** | **Risk difference (95% CI)** |
| --- | --- | --- | --- | --- | --- |
| **No** | 38 (26.4%) |  | Reference | | |
| **Yes, ≥1hour/week** | 64 (43.8%) | 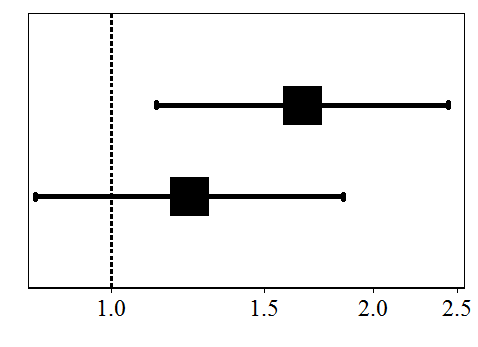 | Crude model  1.66 (1.13, 2.44) | 0.01 | 0.35 (0.28, 0.42) |
|  |  |  | Adjusted model  1.23 (0.82, 1.85) | 0.32 | 0.07 (-0.06, 0.21) |
| **Additional Table 2 Associations between participation in organized sports and likelihood of meeting the physical activity recommendation**  Adjusted model: adjusted for sex, age, overweight/obesity status, accelerometer wear-time, parental education and number of siblings | | | | | |

|  | **Analytical dataset (n=290)** |  | **Excluded observations**† **(n=104)** |
| --- | --- | --- | --- |
| **Descriptive characteristics** |  |  |  |
| Girls, n (%) | 131 (45.2%) |  | 45 (43.3%) |
| age, mean (SD) | 4.7 (0.8) |  | 4.7 (0.7) |
| Overweight/obese, n (%) | 27 (9.3%) |  | 11 (11.6%) |
| Number of siblings, mean (SD) | 1.1 (0.8) |  | 0.9 (0.7) |
| Parental university education, n (%) | 235 (81.0%) |  | 55 (77.5%) |
| **Physical activity whole day, mean (SD)** |  |  |  |
| Moderate to vigorous physical activity (min) | 53.9 (24.3) |  | 52.3 (29.7) |
| Light physical activity (min) | 353.1 (42.7) |  | 342.5 (49.6) |
| Steps (counts) | 9730 (2200) |  | 9275 (2543) |
| Sedentary time (min) | 348.8 (65.7) |  | 349.3 (78.9) |
| Wear-time (min) | 755.9 (56.1) |  | 744.1 (58.4) |
| Meet moderate to vigorous physical activity ≥ 60min/day, n (%) | 102 (35.2%) |  | 38 (36.5%) |
| **Additional Table 3 Comparison of descriptive characteristics between analytical dataset and excluded observations**  †Observations were excluded due to missing data on overweight/obesity status, parental education, number of siblings, report on preschool arrival and departure time and weekend accelerometer measure  Abbreviations: SD = standard deviation | | | |
